# Conserved and reproducible bacterial communities associate with extraradical hyphae of arbuscular mycorrhizal fungi

**DOI:** 10.1038/s41396-021-00920-2

**Published:** 2021-03-01

**Authors:** Bryan D. Emmett, Véronique Lévesque-Tremblay, Maria J. Harrison

**Affiliations:** 1 Boyce Thompson Institute, Ithaca, NY USA; 2 Present Address: USDA Agricultural Research Service, National Laboratory for Agriculture and the Environment, Ames, IA USA; 3 Present Address: Laurentian Forestry Center, Canadian Forest Service, Natural Resources Canada, Quebec City, QC Canada

**Keywords:** Symbiosis, Microbiome

## Abstract

Extraradical hyphae (ERH) of arbuscular mycorrhizal fungi (AMF) extend from plant roots into the soil environment and interact with soil microbial communities. Evidence of positive and negative interactions between AMF and soil bacteria point to functionally important ERH-associated communities. To characterize communities associated with ERH and test controls on their establishment and composition, we utilized an in-growth core system containing a live soil–sand mixture that allowed manual extraction of ERH for 16S rRNA gene amplicon profiling. Across experiments and soils, consistent enrichment of members of the *Betaproteobacteriales*, *Myxococcales*, *Fibrobacterales*, *Cytophagales, Chloroflexales*, and *Cellvibrionales* was observed on ERH samples, while variation among samples from different soils was observed primarily at lower taxonomic ranks. The ERH-associated community was conserved between two fungal species assayed, *Glomus versiforme* and *Rhizophagus irregularis*, though *R. irregularis* exerted a stronger selection and showed greater enrichment for taxa in the *Alphaproteobacteria* and *Gammaproteobacteria*. A distinct community established within 14 days of hyphal access to the soil, while temporal patterns of establishment and turnover varied between taxonomic groups. Identification of a conserved ERH-associated community is consistent with the concept of an AMF microbiome and can aid the characterization of facilitative and antagonistic interactions influencing the plant-fungal symbiosis.

## Introduction

Arbuscular mycorrhizal fungi (AMF) evolved a symbiotic relationship with plants over 400 million years ago and associate with ~72% of all land plants today [1, 2]. The basis of the symbiosis is nutritional and involves the exchange of plant-derived carbon for nutrients, mainly phosphorus and nitrogen, provided by the fungus [3]. The AMF intraradical hyphae access carbon from the root and this fuels growth of extraradical hyphae (ERH) in the surrounding soil, which forage for mineral nutrients essential for fungal growth and for delivery to the plant [4–6]. This symbiosis does not occur in isolation; growth of ERH increases carbon flow into the soil and influences its spatial distribution as the ERH permeate soil micropores inaccessible to roots [7–9]. In the process, ERH interact with soil microbes; NanoSIMs imaging and ^13^C tracing have provided direct evidence that ERH rapidly translocate plant carbon to soil microbes in the process of acquiring mineral nutrients [9–11]. Associations between soil bacteria and ERH are therefore a critical link in the ecology and function of the AM symbiosis in natural and managed ecosystems.

The potential significance of ERH-associated microbial communities can be appreciated in light of insights gained from the genomes of several AMF, which are largely devoid of genes encoding phytases and secreted phosphatases [12, 13] as well as genes required for the degradation of lignin and complex carbohydrates found in plant cell walls [12–17]. This lack of enzymatic capabilities predicts a reliance on soil microbial communities for the release of essential mineral nutrients from complex organic forms. The influence of mycorrhizal ERH on nutrient cycling and to some extent microbial communities has been evaluated using compartmented mesocosms or in-growth mesh cores which exclude roots. These studies documented increased rates of decomposition and nitrogen mineralization from soil and organic residue in the hyphosphere, the zone of soil influenced by the ERH [10, 18, 19]. Meanwhile, shifts in bacterial community composition were revealed using phospholipid fatty acid analysis and 16S rRNA microarrays [10, 19]. Phytate is a dominant form of organic phosphorus in many soils [20] and in-growth cores enriched in phytate showed increased phosphatase and phytase activities around the ERH, and enrichment of alkaline phosphatase producing bacteria associated with the ERH [21–23]. The diverse, positive effects of individual bacteria have long been known [24, 25], but AMF-associated microbial communities are only just beginning to be described [23, 26, 27] and their functions remain to be determined. Importantly, AMF interactions with soil microbial communities are not limited to facilitative or mutualistic responses as soil biota appear to differentially inhibit AMF colonization and activity across diverse soils, whereas in the same steam pasteurized soils such inhibition was not observed [28, 29].

Efforts to investigate microbial communities associated with the ERH face the immense challenge of sampling the delicate and diffuse hyphae from the soil. Consequently, a range of complementary approaches have been devised. In vitro approaches to assess the attachment of individual strains or even complex communities to the ERH have provided evidence for attachment of a subset of the soil bacterial community [30–32]. Furthermore, differential requirements for living or dead hyphae, or for a particular species of AMF, indicate a level of specificity to these interactions [31]. The complementary approach of profiling communities associated with the ERH has so far focused largely on ERH-colonized soil, which may not reveal taxa intimately associated with ERH. Bridging these approaches, artificial soil systems have been used to culture phosphate solubilizing bacteria that attach to the ERH of plants grown in a turface medium [33] and recently, in-growth cores with soil and glass beads [23] have allowed sampling of the ERH-associated community.

Using a modified soil–sand system and gentle rinsing and decanting, ERH can be sampled from a soil–sand matrix and allow high-resolution profiling of ERH-attached communities in a live soil system. Here, in a series of three mesocosm experiments, we detail the bacterial community closely associated with AMF ERH and ask whether this community varies across soils, changes with time, or is influenced by either fungal species or nutrient status of the soil.

## Methods

### Mesocosm design and plant growth conditions

#### Fungal inoculum

Surface-sterilized spores of *Glomus versiforme* (Accession IT104, https://invam.wvu.edu), maintained on leek (*Allium porrum* L.), were prepared as in Liu et al. [34] with minor modifications including sonicating twice in 0.1% Tween-20 for 5 min, surface sterilization by two treatments with 2% Chloramine-T in 0.1% Tween-20 solution on a shaker for 15 min each, followed by 1-h incubation with 200 µg ml^−1^ streptomycin at 4 °C and then rinsed 5x with sterile, deionized H_2_O. Spores from *Rhizophagus irregularis* (DAOM197198) maintained in axenic carrot root organ cultures [35] were harvested by blending the agar spore mix twice for 3–4 s in 20 ml of 10 mM sodium citrate, pH 6.0 and filtering through a 250 µm mesh to trap hyphae and root fragments and a 50 µm mesh to collect spores before resuspending in sterile deionized H_2_O.

#### Experiment 1

A mesocosm experiment was conducted to characterize bacterial communities associated with ERH and test whether these communities differed among background soils. Mesocosms (1.62 l tree pots, Steuwe & Sons, Tangent, OR, USA) consisted of a plant compartment filled with a sterilized sand–gravel mixture and a hyphal in-growth core containing a live soil (Fig. S1).

The plant compartment was filled with an autoclaved mixture of play sand, filter sand, and gravel (2:2:1 v/v/v) [36] with a gently firmed layer of autoclaved play sand (100 ml) at a depth of 8 cm onto which 1000 *G. versiforme* spores were pipetted. A 3 × 16 cm cone placeholder was embedded in the plant compartment to reserve space for the hyphal in-growth core. The mesocosms were planted with ten surface-sterilized seeds of *Brachypodium distachyon* (L.) P. Beauv. line Bd21 and maintained in a growth chamber under 12 h light–dark cycle at 22 °C night and 24 °C day. Pots were fertilized 3x weekly with 50 ml 1/4x modified Hoagland’s solution [37] with 20 µm potassium phosphate.

**Fig. 1:**
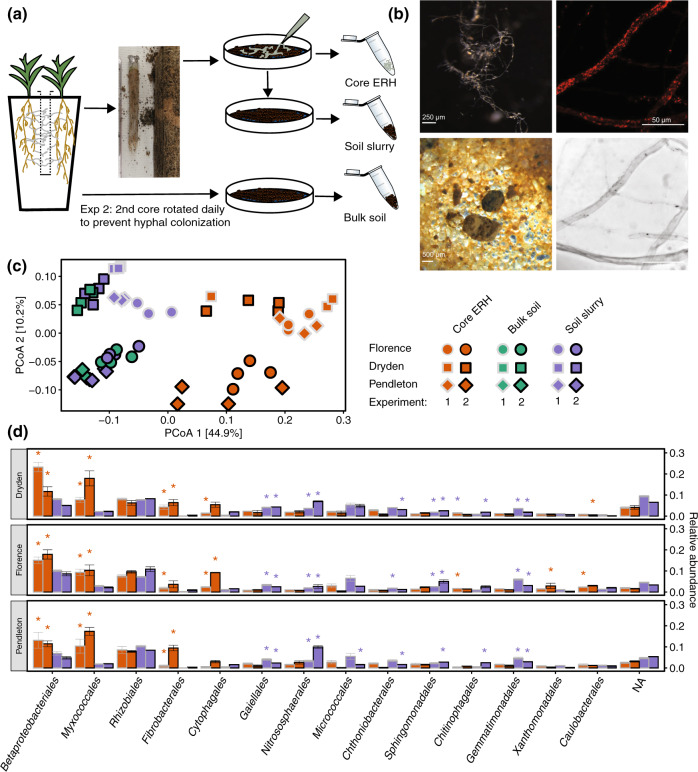
Distinct bacterial communities associate with the ERH of *G. versiforme*. **a** Mesocosm design and sample processing. **b** Clockwise from upper left: Core ERH sample, fluorescent in situ hybridization with universal bacteria probe EUB338-Alexa 594 visualizing bacteria on ERH, brightfield image of ERH, brightfield image of soil slurry sample. **c** Principal coordinate analysis of weighted-UniFrac distances displays variation among sample types and soils. **d** Community composition of ERH samples is consistent across soils and reproducible in multiple experiments. Relative abundance of 15 most abundant bacterial orders in ERH (orange bars) and core slurry samples (purple bars) is arranged by descending abundance in Dryden core ERH samples. Bars represent mean (±SE) of *n* = 3 (Experiment 1), *n* = 8 (Experiment 2, Dryden and Florence), and *n* = 4 (Experiment 2, Pendleton) samples. Bars marked with * are significantly different between ERH and soil slurry samples for the soil and experiment combination (Benjamini–Hochberg adjusted *p* < 0.05).

Forty-nine days after planting, the cone placeholder was removed and the hyphal in-growth core inserted. This two-stage approach was designed to allow the inoculated fungus to colonize the root system and produce a network of ERH, which, supported nutritionally through their connection to the roots, has the capacity to rapidly proliferate in the in-growth core thus outcompeting any native AMF inoculum introduced with the soil core. The in-growth core consisted of a 2.5 × 15 cm mesh core (50 µm, Midwest Filter, St. Charles, IL, USA) containing a live (unsterilized) 2 mm sieved soil mixed with autoclaved play sand (1:1 v/v). Three replicate mesocosms were established for each of three field soils from Dryden, New York (42°31′16″N, 76°19′51″W), Florence, South Carolina (34°18′41″N 79°45′15″W), or Pendleton, South Carolina (34°37′37″N 82°44′27″W). These soils are distinct in texture and carbon content (Table S1). Additionally, mesocosms were established with cores filled only with autoclaved play sand to assess the composition of a community established in the absence of a live soil inoculum (see Supplementary Text and Figs. S2 and S3 for results). Following insertion of the core, ERH were allowed to colonize the core for 84 days before the cores were removed and mesocosms harvested (Figs. 1a and S1).

#### Experiment 2

A second experiment was conducted to confirm that results from Experiment 1 could be replicated and to test the effect of fungal species and soil nutrient status on the composition of the ERH-associated bacterial community. Soil was collected from independent locations within each of the same fields used in Experiment 1, stored at 4 °C, and handled as separate replicates for the duration of the experiment. Mesocosms and experimental conditions were maintained as in Experiment 1, with the following adjustments. Mesocosms included four in-growth cores, two colonized by hyphae and a third rotated 720° daily to prevent hyphal access. A fourth core allowed both root and hyphal access but was not sampled in this experiment. The proportion of the soil component of hyphal in-growth cores was reduced to 1:3 live soil: autoclaved sand (w/w) mixture and the core mesh size reduced to 25 µm. Mesocosms were inoculated with 1000 *G. versiforme* spores. In order to test whether fungal species influenced the ERH community, additional mesocosms slated to receive Dryden soil were inoculated with 3000 *R. irregularis* spores. A nutrient treatment of low phosphorous (1/4x modified Hoagland’s solution with 20 µm potassium phosphate) or low nitrogen fertilizer regime (1/4x Hoagland’s solution with 200 µm potassium phosphate and 1/20x nitrogen) was imposed on mesocosms containing Dryden and Florence soils following core insertion. This resulted in 7 treatment combinations and 28 experiment units (*n* = 4, Table S2). All treatments received nitrogen as NH_4_NO_3_. Mesocosms were harvested 27 days after core insertion.

#### Experiment 3

To characterize the temporal dynamics of community establishment and succession on the hyphae a time course experiment was undertaken using the Dryden soil collected in Experiment 2. Mesocosms of 0.66 l deepots (Steuwe & Sons, Tangent, OR) were inoculated with 300 spores of *G. versiforme* and planted with two seeds of *B. distachyon*. Fewer spores were used as the pot volume was smaller in this experiment. Two cone placeholders were removed 49 days after planting and replaced with 25 µm mesh cores filled with a 1:3 (w/w) mixture of live soil and autoclaved play sand. One core was rotated 720° daily to prevent hyphal access, while hyphae were allowed to access the other core. Pots were fertilized 3x weekly with 25 ml of 1/4x modified Hoagland’s solution with 20 µm potassium phosphate. Six replicate mesocosms were harvested 14, 24, 35, 45, and 65 days after cores were inserted.

### Harvest and sample analysis

The mesh core was removed, cut open vertically with ethanol-rinsed scissors, and the soil–sand mixture placed in a large petri dish. The mixture was gently washed by filling the dish with sterile deionized H_2_O and decanting six times. The hyphal mass was transferred to a fresh petri plate in sterile phosphate-buffered saline (PBS) and hyphae removed from the mass and cleaned of adhering particulate organic matter (POM) using forceps and a 24-gauge needle under a dissecting scope. Hyphae were rinsed by transferring through droplets of sterile PBS in a sterile petri dish and the process repeated until an ERH sample mainly free of POM was collected in a microfuge tube as “core ERH” sample (Fig. 1a, b). In Experiment 1 and later harvests of Experiment 3, some roots had penetrated all hyphal cores. In such cases, hyphae were separated from roots under the dissecting scope and inspected to ensure no root fragments remained as part of the sample.

Additional samples types were collected as controls to identify organisms comparatively enriched on the ERH (Figs. 1a and S1). Following removal of hyphae, a bulb syringe was used to collect a “soil slurry” of the soil–sand matrix under the dissecting scope, providing the primary control for comparison and identification of the ERH-associated community. Additionally, “bulk soil” and “bulk hyphosphere” samples were collected from the rotated core and second intact core, respectively, by homogenizing the soil–sand mixture in a sterile petri plate with an ethanol cleaned spatula and collecting in 2 ml microcentrifuge tubes. Prior to mixing and collection of bulk hyphosphere samples, the cores were inspected to ensure there were no roots present.

It was not possible to completely clear hyphae of all adhering POM (Fig. 1b), therefore, two efforts were made to confirm ERH communities did not reflect POM retained in core ERH samples. First, POM samples were collected from the bulk soil cores following the same process as the core ERH collection—rinsing and decanting with sterile deionized H_2_O six times, then disrupting aggregates with the back of a spatula and collecting remaining POM by decanting the supernatant through a 50 µm nylon filter. The sample was then rinsed with sterile deionized H_2_O and collected in a microfuge tube as “bulk POM” (Fig. S1).

Second, ERH in the root compartment were not in direct contact with the soil matrix, and therefore offered an opportunity to observe the ERH samples in a neutral medium without interference from organic matter. Roots were rinsed in ddH_2_O and floated in sterile PBS for collection of “root ERH” samples. As a control for comparison with root ERH, a subsample of the sand–gravel matrix in the root compartment was collected in Experiment 2, rinsed with ddH_2_O, cleared of hyphae under a dissecting scope and a “sand slurry” collected in a bulb syringe. All samples were immediately flash frozen in liquid nitrogen for downstream analysis of nucleic acids.

### 16S rRNA gene sequence analysis

DNA was extracted from samples and dual-barcoded MiSeq libraries of 16S rRNA V4 gene amplicons were prepared and sequenced on the Illumina MiSeq platform. Demultiplexed files from each library were processed separately with the “DADA2” pipeline in R [38] to trim, filter, and infer amplicon sequence variants (ASVs) in each sample, remove chimeras, and construct a sequence count table. The count table, phylogenetic tree, taxonomy table, and sample metadata were combined in a “phyloseq” object for further analysis [39]. Details of library preparation, sequence processing, and phylogenetic tree construction are summarized in Table S2 and detailed in Supplementary Text. ASVs unclassified at the phylum level were removed from analysis, which also removed three sequences matching Mollicutes/*Mycoplasma*-related endobacteria (MRE) of *G. versiforme* from the analysis [13], avoiding skewed relative abundance estimates resulting from their high abundance in the hyphal samples. The final data set contained 18 876 ASVs. Sequence counts were checked to ensure similar distribution of reads between treatment groups within an experiment and analysis proceeded using non-rarefied count tables [40]. Raw sequence files and associated metadata were deposited in the NCBI sequence read archive under accession #PRJNA644936. Final phyloseq objects are available in Supplementary Data File 2.

### Statistical analysis

All statistical analyses were conducted in R 3.6.0 [41]. Beta-diversity among samples was calculated using weighted-UniFrac distances [42] on bacterial ASVs only and visualized using principal coordinate analysis (PCoA) in the phyloseq package. Treatment and sample type effects on beta-diversity were assessed using permutational multivariate analysis of variance using the ADONIS function in the vegan package [43]. Identification of ERH-associated taxa was conducted through comparison with soil slurry samples. This approach focuses on taxa specific to, or enriched on, the hyphal samples rather than background soil populations observed across sample types. The approach also limits the confounding influence of roots that penetrate the core as the soil slurry samples experience the same experimental conditions as the hyphae. Sample type influence on natural log-transformed relative abundance of the 15 most abundant prokaryotic orders was assessed in linear models with soil, experiment, and sample type as fixed effects and pairwise comparisons of sample types within a soil and experiment assessed in the package “emmeans” using Benjamini–Hochberg adjusted *p* values [44]. Differential abundance of bacterial and archaea ASVs between sample types was determined using a negative binomial model in the DESeq2 package [45]. Hyphal-associated ASVs were identified as those with log_2_-fold change > 0 (adjusted *p* < 0.05) between core ERH and soil slurry samples in any soil (Supplementary Data File 1). To determine if ERH colonization of the bulk hyphosphere was correlated with the abundance of hyphal ASVs, correlation between MRE relative abundance in the untrimmed data set and relative abundance of groups of hyphal ASVs was tested in a linear model with soil type, replicate, and sample type used as covariates. R code used for sequence processing, statistical analysis, and figure generation are available at https://github.com/bdemmett/Hyphosphere.

## Results

**Fig. 2:**
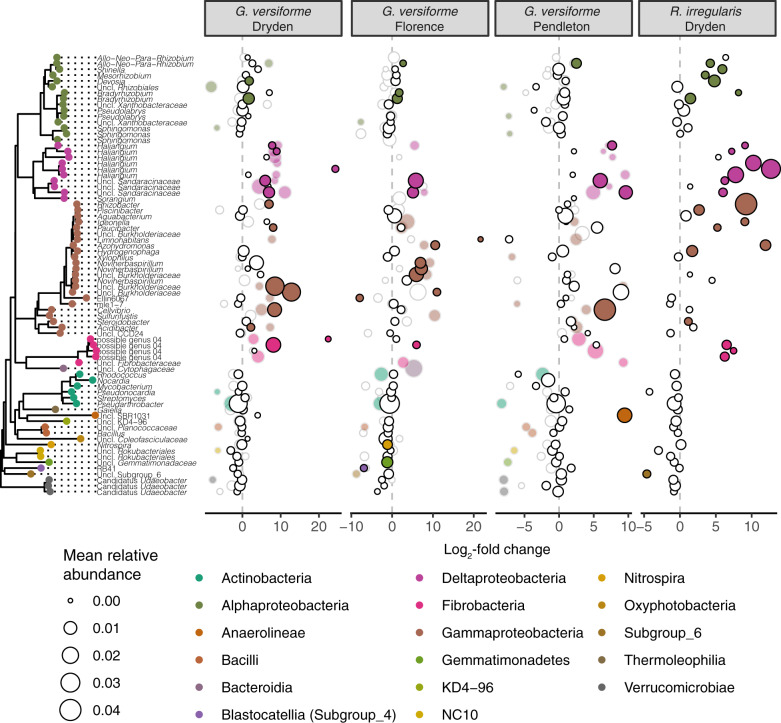
ERH-associated communities of *G. versiforme* are largely conserved at the family and order levels across three soils and between fungal species. Differential abundance of ASVs between core ERH and soil slurry samples in three soils in Experiment 1 (gray outlines, transparent fill; *n* = 3) and Experiment 2 (black outlines, solid fill; *n* = 8 for Dryden and Florence soils and *n* = 4 for Pendleton). ASVs with significant log_2_-fold change (DESeq2: adjusted *p* < 0.05) are colored by taxonomic class. Nonsignificant points have white fill. Taxa organized by phylogenetic tree (left) and log_2_-fold change between core slurry and hyphal samples indicated by position on *x*-axis. Point size indicates mean relative abundance across soil slurry and core ERH samples. Note *R. irregularis* included only in Experiment 2.

16S rRNA gene profiling revealed a distinct bacterial community associated with ERH of *G. versiforme*. Core ERH samples from Experiments 1 and 2 clearly separated from the soil slurry and bulk soil samples in the primary axis of the PCoA of weighted-UniFrac distances (Fig. 1c) and sample type accounted for roughly 40% of the variation among samples from the three soils (Table 1; *p* < 0.001). Across experiments and soils, consistent and taxonomically coherent selection was evident in samples of ERH. Hyphal samples were dominated by *Proteobacteria* (50% relative abundance ± 11% SD), with lesser amounts of *Actinobacteria* (10 ± 5%), *Chloroflexi* (9 ± 6%), *Acidobacteria* (7 ± 2%), *Bacteroidetes* (6 ± 4%), and *Fibrobacteres* (4 ± 4%). At the order level, hyphal samples showed a marked increase in relative abundance of *Betaproteobacteriales, Myxococcales, Fibrobacterales*, and *Cytophagales* compared to the soil slurry (Fig. 1d). These changes followed the enrichment of 298 ASVs in hyphal samples (*p* < 0.05) (Fig. 2), which were largely within the same orders. Several ASVs within *Alphaproteobacteria* and *Gammaproteobacteria* outside of the *Betaproteobacteriales* were also enriched in ERH samples (Fig. 2), but these groups were less consistently enriched across soils and the overall abundance did not increase in ERH samples (Fig. 1d).

**Table 1:** Variance partitioning of bacterial community beta-diversity in Experiments 1 and 2 as measured by weighted-UniFrac distances among sample types, soils, and experimental runs in permutational multivariate analysis of variance.

	df	SS	MS	F	*R*^2^	*p*
Soil	2	0.46	0.23	12.35	0.14	<0.001
Sample type (St)^a^	2	1.31	0.66	35.07	0.40	<0.001
Experiment	1	0.26	0.26	13.85	0.08	<0.001
Soil × St	4	0.16	0.04	2.08	0.05	0.011
St × experiment	1	0.09	0.09	5.07	0.03	0.002
Soil × experiment	2	0.27	0.13	7.12	0.08	<0.001
Residuals	41	0.77	0.02		0.23	
Total	53^b^	3.32			1	

Soils: Dryden, Florence, Pendleton.

^a^Sample types include soil slurry, bulk soil, and core extraradical hyphae.

^b^Only samples from low phosphorous mesocosms included in PERMANOVA to allow comparison across experiments.

Background soil influenced community composition in both ERH samples and bulk soil and soil slurry controls, yet the taxonomic selection evident in ERH-associated communities was consistent across soils. Communities from different source soils separated on the second axis of the ordination and accounted for 14% of the variation in weighted-UniFrac distances (Table 1, *p* < 0.001). Soils separated mainly by biogeography, as the Pendleton, SC and Florence, SC soils, clustered more closely together compared to the Dryden, NY soil. The strength of the soil effect varied between experiments, with greater separation between soils in Experiment 2 (Fig. 1). Nevertheless, the reproducibility of the hyphal community was high, with only 5% of the variation accounted for by the interaction of soil and sample type and only 3% of variation attributed to the interaction of sample type and experimental run (Table 1, *p* = 0.011 and *p* = 0.002, respectively). The ERH-associated community was largely conserved at the family and order levels across soils with variation expressed at lower taxonomic ranks. Enrichment of individual hyphal ASVs was mostly specific to a particular soil and experimental run (Fig. 2). However, a number of ASVs were enriched in multiple soils from both experiments, including ASVs within the *Myxococcales, Sandaracinaceae*, and *Haliangiaceae* families, the *Burkholderiaceae* and a *Cellvibrio* ASV (Fig. 2).

Additional controls were included in Experiment 2 to test whether the hyphal extraction technique contributed to experimental artifacts and confirm that the taxonomic shifts observed in hyphal samples were robust (Fig. S4). The bulk soil and soil slurry samples provided controls that were not colonized by ERH or were processed similarly to the hyphal samples and could be used to evaluate hyphal community composition. Both controls clustered closely together, indicating that sample processing was not driving the observed variation in community composition. Additionally, of the 221 ASVs enriched in hyphal samples of *G. versiforme*, only 34 were also enriched when comparing the POM samples with the same soil slurry. Moreover, a similar taxonomic profile of differentially abundant taxa was established when comparing hyphal samples directly to POM samples (Fig. S5). Thus, most hyphal-associated ASVs were unlikely to be identified due to adhering POM and the majority were not organic matter generalists. Observed generalist ASVs included several *Rhizobiales*, *Chloroflexales*, a *Cellvibrio*, and other phylogenetically diverse taxa.

Samples of ERH collected from the root compartment were evaluated to determine whether the ERH-associated community would establish in a neutral medium. The composition of root ERH was consistent with the family and order level taxonomic selection observed in the hyphae extracted from the soil cores (Fig. S6). Only 41 ASVs were differentially abundant in the root ERH samples (adjusted *p* < 0.05) compared to the sand slurry, possibly a result of roots and hyphae providing the primary carbon source and driving community composition in this compartment. Similar to the core ERH, differentially abundant ASVs were drawn from the *Myxococcales*, *Fibrobacterales*, *Cellvibrionales*, *Chloroflexales*, and *Betaproteobacterales* (Fig. S5).

Comparison of bulk hyphosphere with bulk soil allowed further confirmation that sample processing was not producing experimental artifacts. This comparison was less sensitive than direct extraction of ERH in identifying hyphal-associated taxa. Only a few ASVs were differentially abundant between bulk hyphosphere and bulk soil (log_2_-fold change > 0, adjusted *p* < 0.05), but included previously identified *Burkholderiaceae*, *Sandaracinaceae*, *Herpetosiphon* (Chloroflexi), and *Acidibacter* (*Gammaproteobacteria*) ASVs. However, a priori knowledge of the ERH-associated community could be confirmed in an undisturbed system when controlling for the extent of ERH colonization of the in-growth core using the relative abundance of three sequences from Mollicutes/*Mycoplasma*-related endosymbionts of *G. versiforme* [13]. Grouped by order, hyphal ASVs from the *Myxococcales* and *Fibrobacterales* were both at higher abundance in hyphosphere soils and positively correlated with MRE relative abundance (Fig. S5). Hyphal ASVs from the *Betaproteobacteriales* were also more abundant in hyphosphere soils. In contrast, the more generalist *Chloroflexales* and *Rhizobiales* were not at higher relative abundance in hyphosphere samples or correlated with MRE abundance (Fig. S7).

### Fungal species and fertility treatments

**Fig. 3:**
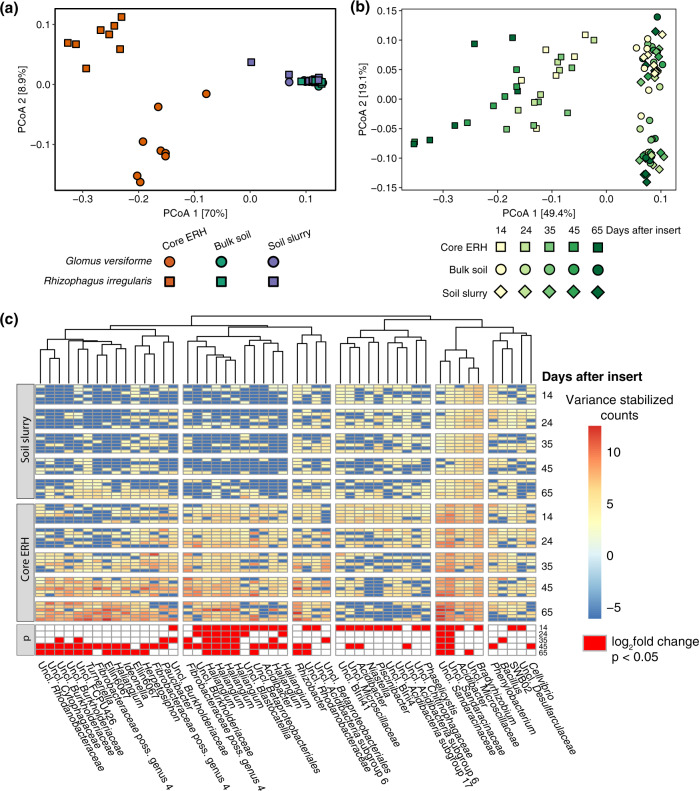
Fungal species and temporal dynamics influence composition of ERH-associated bacterial communities. Principal coordinate analysis of weighted-UniFrac distances displays variation among sample types and fungal species (**a**) and over time (**b**) in Dryden soil. Heatmap of sequence counts, following DESeq2 variance stabilization transformation, displaying variation in ASV abundance over time (**c**). Differential abundance tests for each time point shown below. Shown for clarity: 50 most abundant ASVs identified as significantly enriched in core ERH samples during at least one time point.

Comparison of the ERH-associated communities of *R. irregularis* and *G. versiforme* revealed that the ERH-associated community was conserved, with differences observed between species in the strength and specificity of selection. In the Dryden soil, where both *R. irregularis* and *G. versiforme* hyphal samples were analyzed, fungal species accounted for 5% of the variation among samples with an additional 8% of the variation explained by the interaction of fungal species with sample type (Fig. 3 and Table 2; *p* < 0.001). *R. irregularis* appeared to exert stronger selection on the hyphal communities, resulting in 236 ASVs enriched in *R. irregularis* ERH samples compared to 88 ASVs enriched in *G. versiforme* samples from the same Dryden soil (Fig. 2). The taxonomic profile of the two fungi was similar, but with greater enrichment of *Alphaproteobacteria* and *Gammaproteobacteria* in samples of *R. irregularis*.

**Table 2:** Variance partitioning of bacterial community beta-diversity as measured by weighted-UniFrac distances among sample types and fungal species (*G. versiforme* and *R. irregularis*) in permutational multivariate analysis of variance.

	df	SS	MS	F	*R*^2^	*p*
Replicate block	3	0.08	0.03	2.83	0.03	0.015
Sample type (St)^a^	2	1.55	0.77	82.91	0.68	<0.001
Fungus	1	0.1	0.1	11.11	0.05	0.002
St × fungus	2	0.18	0.09	9.43	0.08	<0.001
Residuals	39	0.36	0.01		0.16	
Total	47	2.27			1	

^a^Sample types include soil slurry, bulk soil, and core extraradical hyphae samples. All samples taken from Dryden soil.

In contrast to the strength of sample type and fungal species, there was no significant effect of modulating the fertilization regime. Hyphae from mesocosms fertilized with either low phosphorous or low nitrogen modified Hoagland’s solutions were not significantly different in weighted-UniFrac distances (*p* = 0.308; Fig. S8).

### Temporal patterns

In a time-series experiment, hyphal in-growth cores were sampled between 14 and 65 days after insertion to identify temporal patterns of establishment or succession in the observed community. A distinct ERH-associated community was established by 14 days and became progressively more distinct from bulk soil and soil slurry samples over time (Fig. 3b) as the portion of reads from hyphal ASVs increased from 17 ± 2% at 14 days to 40 ± 4% at 65 days after insert. Overall, temporal variation accounted for 8% of the variance in weighted-UniFrac distances, while the interaction with sample type explained an additional 9% (Table 3; *p* < 0.001). *Myxococcales* ASVs typified early and consistently enriched hyphal responders. Of 105 ASVs enriched in ERH samples from at least one time point, only 6 ASVs from the *Sandaracinaceae* and *Haliangiaceae* families (order *Myxococcales*) were significantly enriched at all sampling dates. Other groups established early but decreased in abundance over time, including ASVs from the BIrii41 family, some *Acidibacter* and *Dokdonella* (*Gammaproteobacteria*) (Fig. 3c). Members of the *Betaproteobacteriales* were also early colonizers of the hyphal samples, but there was turnover in this group as individual ASVs decreased in abundance and others increased in abundance over time. Meanwhile, *Cytophagales, Chloroflexales*, and *Fibrobacterales* increased in abundance over time.

**Table 3:** Variance partitioning of bacterial community beta-diversity among sample types and sampling date in permutational multivariate analysis of variance.

	df	SS	MS	F	*R*^2^	*p*
Replicate block	5	0.42	0.08	5.72	0.12	<0.001
Sample type^a^	2	1.48	0.74	50.05	0.42	<0.001
Days after insert (DAI)^b^	4	0.27	0.07	4.58	0.08	<0.001
Sample type × DAI	8	0.31	0.04	2.61	0.09	<0.001
Residuals	68	1.01	0.01		0.29	
Total	87	3.49			1	

^a^Sample types include soil slurry, bulk soil control, and core extraradical hyphae samples. All samples taken from Dryden soil.

^b^In-growth cores harvested 14, 24, 35, 45, and 65 days after insert.

## Discussion

In a series of mesocosm experiments, we have detailed a conserved and reproducible bacterial community associated with ERH of two AMF species during symbiosis with *B. distachyon*. Repeated enrichment for members of the *Betaproteobacteriales*, *Myxococcales*, *Fibrobacterales*, *Cytophagales*, *Chloroflexales*, and *Cellvibrionales* was observed in ERH samples across experiments and divergent soils. ASVs from other groups, such as the *Alphaproteobacteria* and *Gammaproteobacteria*, were also enriched in hyphal samples, but were more variable across soils and did not achieve higher abundance as a group. Within this taxonomic structure, there was a smaller but notable variation observed at the level of individual ASVs as few ASVs were enriched across all soils. Similar taxonomic selection was observed in both fungal species assayed.

The concept of an AMF-associated microbiome has largely been discussed in relation to endobacteria inhabiting the cytoplasm or membrane-bound compartments of many AMF species [46–48]. While endobacteria represent the closest association of AMF and bacteria, the results presented here on recurring associations at the ERH surface may also be viewed in the context of an AMF microbiome. In this context, it appears there is a core microbiome associated with the ERH of AMF, shaped by selection at higher taxonomic ranks, but with expressed variation among soils. This dynamic is similar to plant root and rhizosphere microbiomes, where broad selection for particular phylogenetic groups is consistent across sites and expression of unique microbiome composition likely arises in response to site-specific constraints or microbe–microbe interactions [49–51].

The *Betaproteobacteriales*, formerly the *Betaproteobacteria* [52], are frequent and significant components of plant microbiomes [49, 53] and our results add to previous reports of their interaction with AMF. *Betaproteobacteriales* interaction with AMF range from the vertically transmitted endosymbionts known as *Burkholderia*-like organisms (*Candidatus* *Glomeribacter gigasporarum*) [17, 47, 48, 54] to more broad fungal-associated lifestyles. In vitro attachment of *Betaproteobacteria* to ERH was observed by Scheublin et al. [32], who found *Oxalobacteraceae* were overrepresented in hyphal attached communities. Similarly, Offre et al. [55, 56] found that *Oxalobacteraceae* were preferentially associated with mycorrhizal roots as compared to non-mycorrhizal roots. In this study, hyphal ASVs from the *Betaproteobacteriales* were mainly within the *Burkholderiaceae* and primarily consisted of taxa formerly classified as *Comamonadaceae* but included some formerly classified as *Oxalobacteraceae* [52]. The *Burkholderiaceae* are significant producers of exo- and lipopolysaccharides in soil [57] and bacterial biofilm formation on ectomycorrhizal fungal hyphae appears to be widespread [58]. This capability could be a mechanism of attachment to the ERH surface and also contribute to mycorrhizal impacts on soil aggregation and structure in the field [59, 60]. Additionally, several taxa display antifungal activity, including chitinolytic genera [61, 62]. Turnover of ERH in the field is rapid [63] and chitinolytic organisms associated with ERH may be poised to benefit from the input of fungal biomass.

A striking observation in our data sets is the consistent enrichment of putative bacterial predators in hyphal samples. The *Myxococcales*, a group known for their predatory and social behavior [64], were universally enriched across soils and strong and consistent colonizers throughout the time course experiment. Several other hyphal ASVs are also known bacterial predators, including *Herpetosiphon* (*Chloroflexales*), *Cytophagaceae* ASVs (*Cytophagales*), *Bdellovibrio* (*Bdellovibrionales*), and a *Micavibrionales* ASV [65]. The strong increase in abundance of bacterial predators could indicate changing trophic structure in the hyphosphere and contribute to the mineralization of nutrients from microbial biomass. From the predator standpoint, if the surface of ERH are enriched in bacterial populations, the hyphae may serve as linear feeding lanes. Alternately, it cannot be ruled out that these organisms are antagonistic to the fungi as several have been noted to prey on fungi or inhibit fungal activity [66, 67]. Though to our knowledge there is no evidence for myxobacteria grazing on AMF.

Beyond predatory lifestyles, many of the bacterial predators and other ERH-associated taxa are well equipped with hydrolytic enzymes for breaking down complex substrates [68]. Similar to rhizosphere dynamics, such organisms may utilize fungal exudates to subsidize the production of expensive extracellular enzymes. In a recent metagenome resolved transcriptomic study, both *Burkholderiaceae* and *Fibrobacteria* upregulated transcription of carbohydrate active enzymes 3x greater in combined rhizosphere and detritosphere habitats than when exposed to either substrate alone [69]. These groups are candidates for further investigation for their contribution to observed hyphosphere priming of carbon and nitrogen mineralization [19, 70, 71].

### Fungal species

While sharing a broad taxonomic profile, *R. irregularis* and *G. versiforme* differed in the strength and specificity of selection on the hyphal-associated community. Host species selection on microbiome composition is frequently observed in mammalian, plant, and invertebrate systems [72–75]. *R. irregularis* and *G. versiforme* diverged ~331 Ma years ago [13] and therefore it is not surprising that variation in traits shaping their associated microbiota would evolve. These findings are consistent with previous reports of variation among AMF lineages in their associated microbiota following long-term co-cultures with the same host plant [76] and observations that bacterial strain ability to attach to and colonize ERH in vitro depended on the fungal species assayed [31]. The traits driving this selection remain unexplored. Fungal exudates have been noted to influence soil bacterial community composition and metabolism of associated bacteria [77, 78]. If AMF species vary in hyphal exudates, metabolites, or signaling that drives changes in the composition and activity of hyphal-associated bacterial communities, such variation could have important implications for nutrient cycling in soil and contribute to functional variation among AM symbioses.

### Nutrient status

In contrast to earlier reports, modulating nutrient availability [23, 79] did not alter the composition of the ERH-associated community. This conflict may result from the type of fertilizer and its impacts on the physiochemical environment. Toljander et al. [79] found that fertilizer-driven shifts in the mycorrhizosphere community were largely correlated with pH, a well-known determinant of bacterial community composition [80, 81]. We did not measure pH in the mesocosms, but regular application of the liquid Hoagland’s solution may have limited variation between the treatments. Additionally, inorganic and soluble forms of both nitrogen and phosphorous were used, potentially limiting selection for nutrient specialists.

### Temporal trends

A time course of sampling, 14–65 days after in-growth cores were inserted, allowed us to observe the dynamic establishment of the hyphal attached community. These temporal dynamics were largely consistent with variation between Experiments 1 and 2, when the in-growth cores were in place for 87 and 27 days, respectively. Fewer ASVs from the *Betaproteobacteriales* were enriched in hyphal samples from Experiment 2, along with a lower abundance of *Cytophagales*, *Fibrobacterales*, and *Chloroflexales* (Figs. 2 and 3). These same groups showed a strong increase in abundance over time in Experiment 3. Similar turnover of rhizosphere bacterial communities has been observed and linked with changes in exudate profiles of the host plant during development [82, 83]. Whether metabolic changes in the hyphal network manifest over time as the host plant growth slows or whether differences in bacterial growth rates or microbe–microbe interactions, such as the increased abundance of bacterial predators, explain the dynamics we observed is an open question.

## Conclusion

AMF play a critical role in plant nutrient acquisition and are looked to as an important strategy to support ecological intensification of agricultural systems [84]. To fulfill that promise, improved understanding of the ecology of AMF, including their interaction with other soil biota, will be critical. We propose that the hyphal-associated community detailed here reflects a broad adaptation among certain bacterial groups to a fungal-associated lifestyle and suggest that refinement of this community occurs among fungal species and soils as individual strains interact with the fungi and compete in a given environment. Identification of this AMF-associated microbiota can guide future efforts to understand the fungal–bacterial interactions that give rise to this community and identify organisms beneficial or antagonistic to AMF in the field. Broadening this analysis to the genes and functions of the AMF-associated microbiome and interrogation of AMF interactions with other eukaryotic soil biota will be necessary to fully appreciate the ecology of these important plant symbionts.

## Supplementary information

Emmett et al Supplementary materials

Supplementary Data File 1

Supplementary Data File 2
