# Supplementary material for: Conserved and reproducible bacterial communities associate with extraradical hyphae of arbuscular mycorrhizal fungi
Source: ISME J. 2021 Mar 1;15(8):2276–88. doi: 10.1038/s41396-021-00920-2 (PMC8319317; doi:10.1038/s41396-021-00920-2)
Supplement: Supplementary file 1 — Emmett et al Supplementary materials [file 41396_2021_920_MOESM1_ESM.pdf]

Conserved and reproducible bacterial communities associate with extraradical hyphae of arbuscular mycorrhizal fungi

Emmett, Bryan D; Lévesque-Tremblay, Véronique; Harrison, Maria J

Boyce Thompson Institute, 533 Tower Road, Ithaca, NY 14853, USA

Authors for contact: mjh78@cornell.edu and be68@cornell.edu

Present Addresses:
B.D. Emmett, USDA Agricultural Research Service, National Laboratory for Agriculture and the Environment, 1015 N. University Blvd. Ames, IA 50011. (Email: bryan.emmett@usda.gov)
V. Lévesque-Tremblay, Laurentian Forestry Center, Quebec, Canada

Supplementary Information

Table S1: Characteristics of source soils included in mesocosm experiments

Soil	pH	Al*	Ca	Cu	Fe	PK	Mg	Mn	Mo	Na	P	S	Zn	TC[a]	TN[b]
		mg/Kg	mg/Kg	mg/Kg	mg/Kg	mg/Kg	mg/Kg	mg/Kg	mg/Kg	mg/Kg	mg/Kg	mg/Kg	mg/Kg	%	%
Dryden	6.26	8.39	704.2	0.03	0.58	39.87	59.63	6.02	0.00	12.24	6.23	3.47	0.11	2.25	0.26
Florence	6.39	5.57	234.2	0.02	0.44	13.90	12.58	1.17	0.00	12.44	7.05	2.50	0.56	0.73	0.11
Pendleton	5.73	11.13	161.3	0.04	0.31	11.45	30.69	6.59	0.00	10.84	0.19	9.80	0.13	1.00	0.11

	Sand	Silt	Clay
Dryden	44	43	13
Florence	78	17	5
Pendleton	46	15	38

*Modified Morgan nutrient analysis (Al – Zn)

[a] Total carbon

[b] Total nitrogen

Table S2: Experiment set up and library preparation details for three mesocosm experiments

Exp.	Pot size	Plants	Spores	Cores	Mesh size	Core substrate	Core insert (Days after planting)	Harvest (Days after insert)	Treatments and replication					
									Soil	Fungi	Nutrient	Time	n	Total
1	1.62 l	10	1000 *G. versiforme*	1	50 µm	1:1 soil: sand (v/v)	49	84	Dryden	1	1	1	3	12
									Florence	1	1	1	3	
									Pendleton	1	1	1	3	
									Sand	1	1	1	3	
2	1.62 l	8	1000 *G. versiforme*, 3000 *R. irregularis*	4	25 µm	1:3 soil: sand (w/w)	49	27	Dryden	2	2	1	4	28[a]
									Florence	1	2	1	4	
									Pendleton	1	1	1	4	
3	0.66 l	2	300 *G. versiforme*[b]	2	25 µm	1:3 soil: sand (w/w)	49	14, 24, 35, 45, 65	Dryden	1	1	5	6	30

Exp.	Sample types	DNA extraction	Library preparation	Sequencing
1	Core ERH, soil slurry, root (mycorrhizosphere)	Qiagen Powersoil[c]	University of Minnesota Genomics Center	University of Minnesota Genomics Center - Illumina MiSeq v3
2	Bulk soil, core ERH, soil slurry, bulk hyphosphere, particulate organic matter, root ERH, sand slurry	Phenol:Chloroform [1]	Dual barcoded (Kozich et al. 2013)	Cornell Biotechnology Resource Center Genomics Facility (Ithaca, NY, United States) - Illumina MiSeq v2
3	Bulk soil, core ERH, soil slurry, particulate organic matter, root ERH, root (mycorrhizosphere)	Qiagen PowerMicrobiome	Dual barcoded (Kozich et al. 2013)	Cornell Biotechnology Resource Center Genomics Facility (Ithaca, NY, United States) - Illumina MiSeq v2

[a]An additional 8 *R. irregularis* mesocosms were established in Florence soil but were only sampled for bulk soil and bulk hyphosphere and are not included in the analysis, but are available in Supplementary Data File 2.
[b] Inoculum level adjusted for size of mesocosm
[c]Hilden, Germany

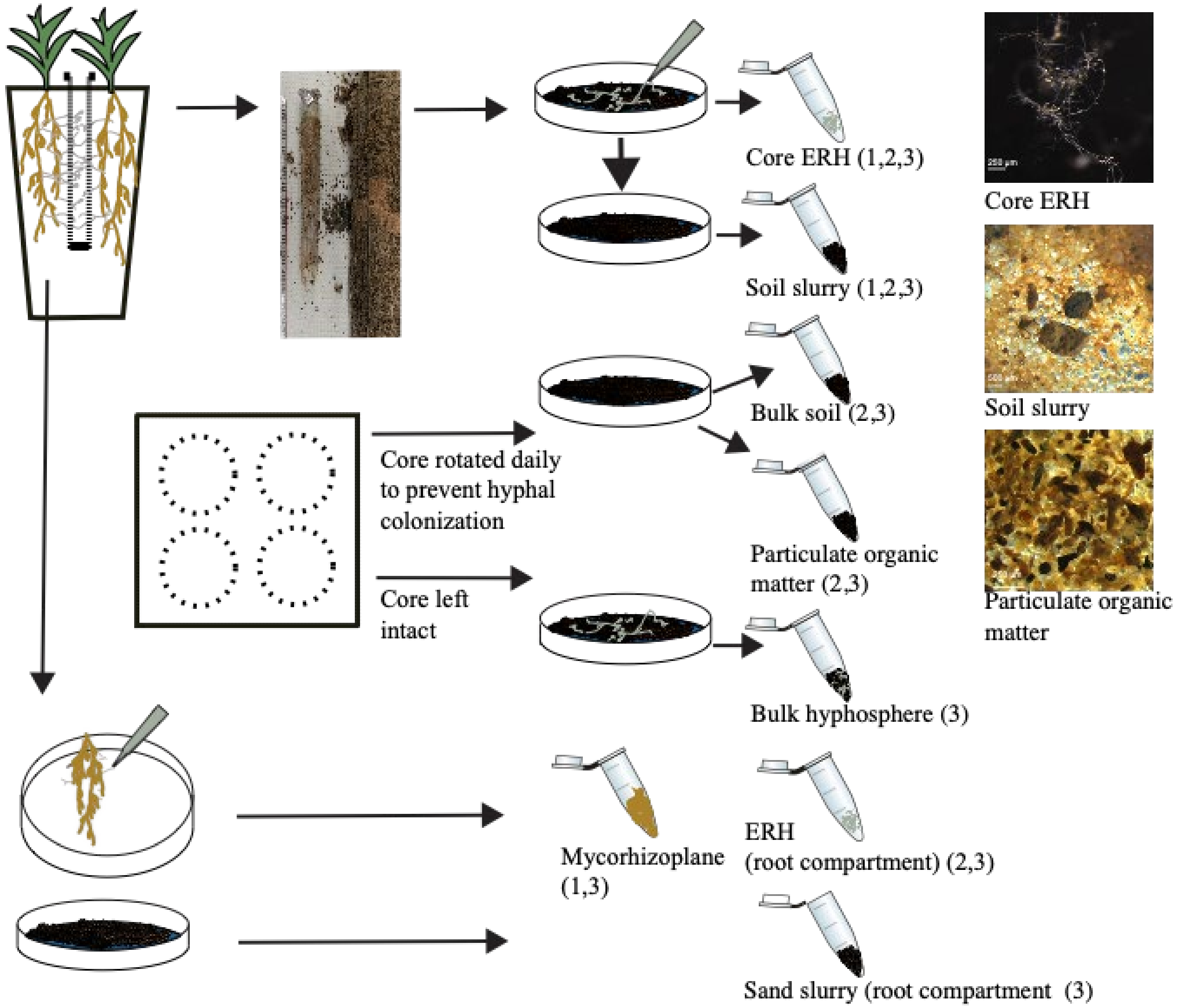

Figure S1: Expanded sample harvesting from mesocosms. Numbers in parentheses indicate each experiment for which the sample type was collected.

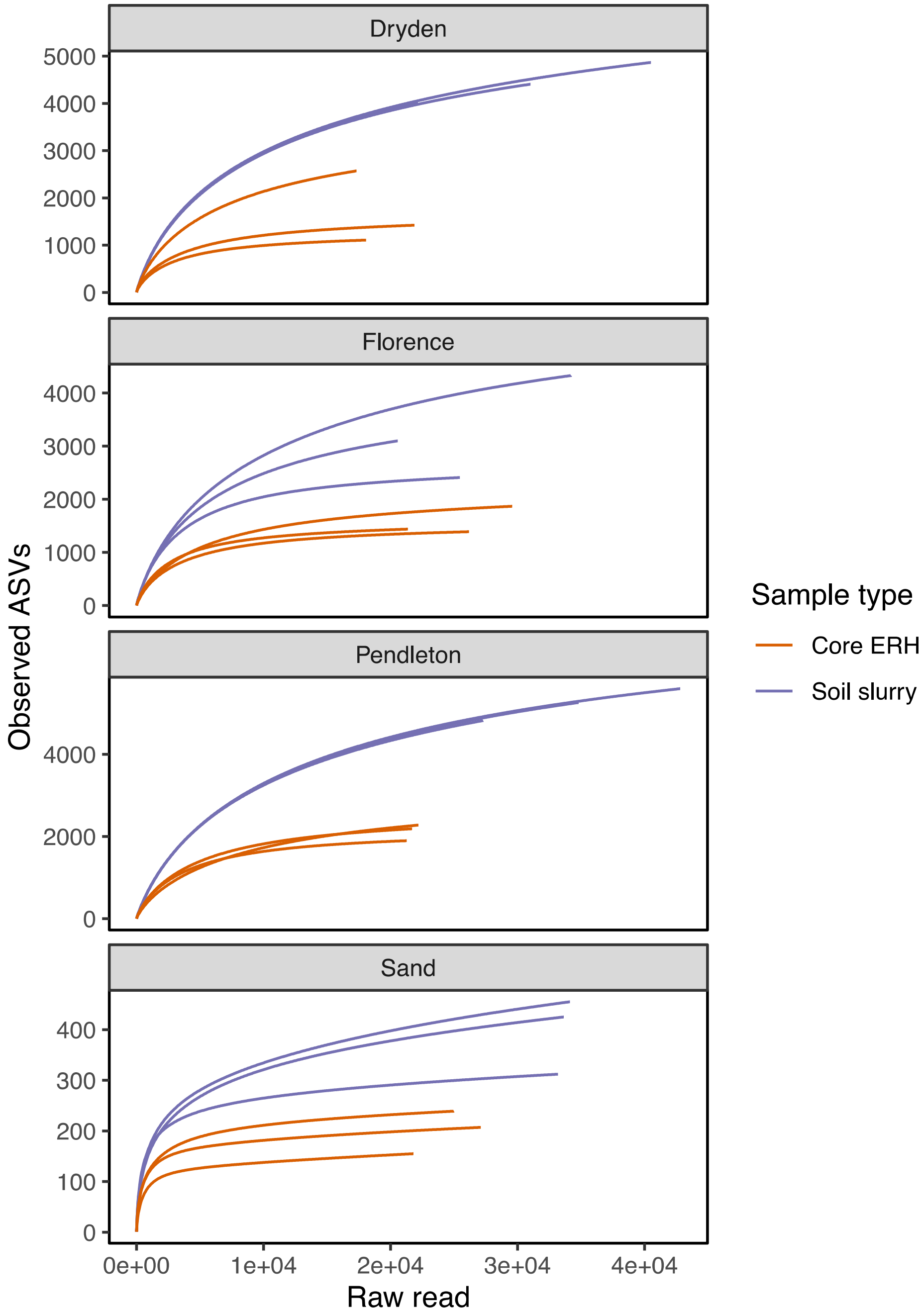

Figure S2: Rarefaction curves from soil slurry and core extraradical hyphae (ERH) samples in Experiment 1. Rarefaction curves were generated from separately processed sequences using the pool = TRUE option in DADA2 to retain singletons and doubletons in samples and allow evaluation of sampling depth. Main analysis conducted on dataset processed sample-wise for computational efficiency.

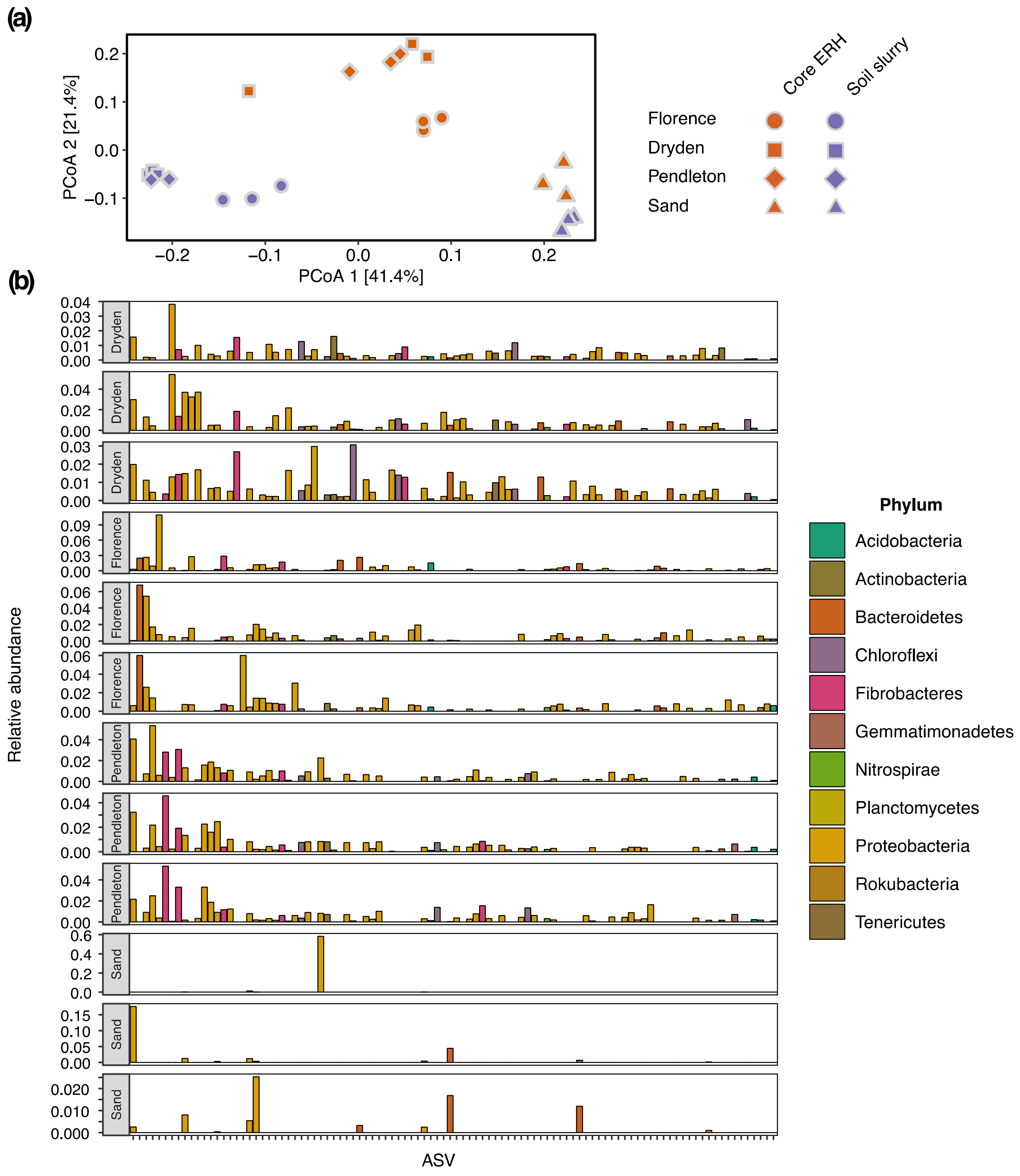

Figure S3: Community composition of sand controls compared to live soil mesocosms. (a) Principal coordinate analysis shows separation of samples collected from sterilized sand in-growth cores and samples collected from live soil in-growth cores. (b) 100 most abundant taxa on core ERH from live soil cores in Experiment 1 are only sporadically identified on ERH from sterilized sand controls. Facets display relative abundance on ERH from individual replicates of mesocosms receiving live soil cores (top nine facets) or sterilized sand cores (bottom three facets). Patterns are consistent with occasional colonization by taxa from live soil controls as mesocosms were not maintained under aseptic conditions. Note variation in scale of y-axis.

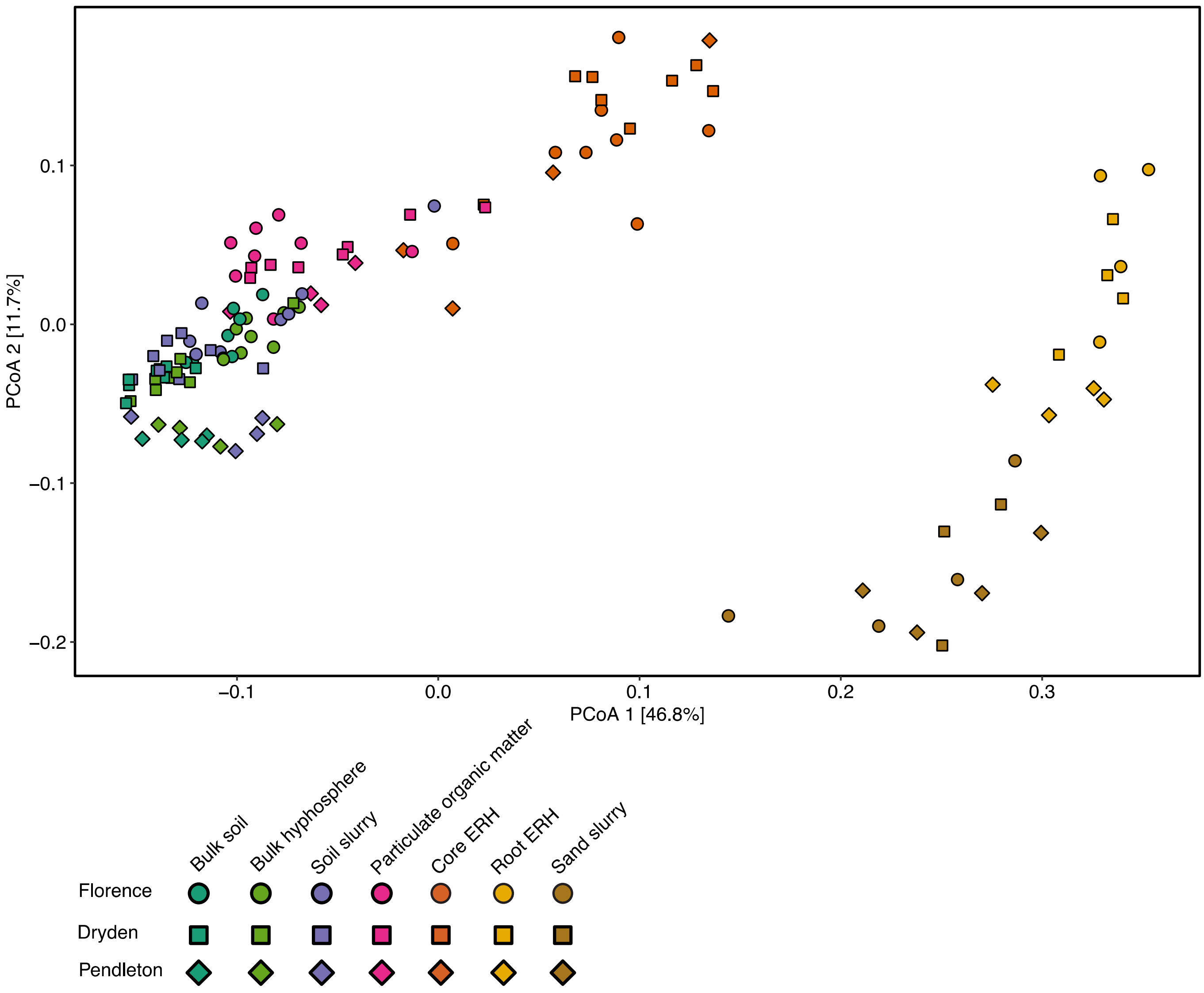

Figure S4: Principal coordinate analysis of weighted-Unifrac distances displays variation among sample types in Experiment 2. Core ERH samples separate from particulate organic matter and bulk soils on both the primary and secondary axis. ERH from the root compartment show similar separation from the sand slurry samples in the same compartment, but both sample types are distinct from samples taken from the soil core.

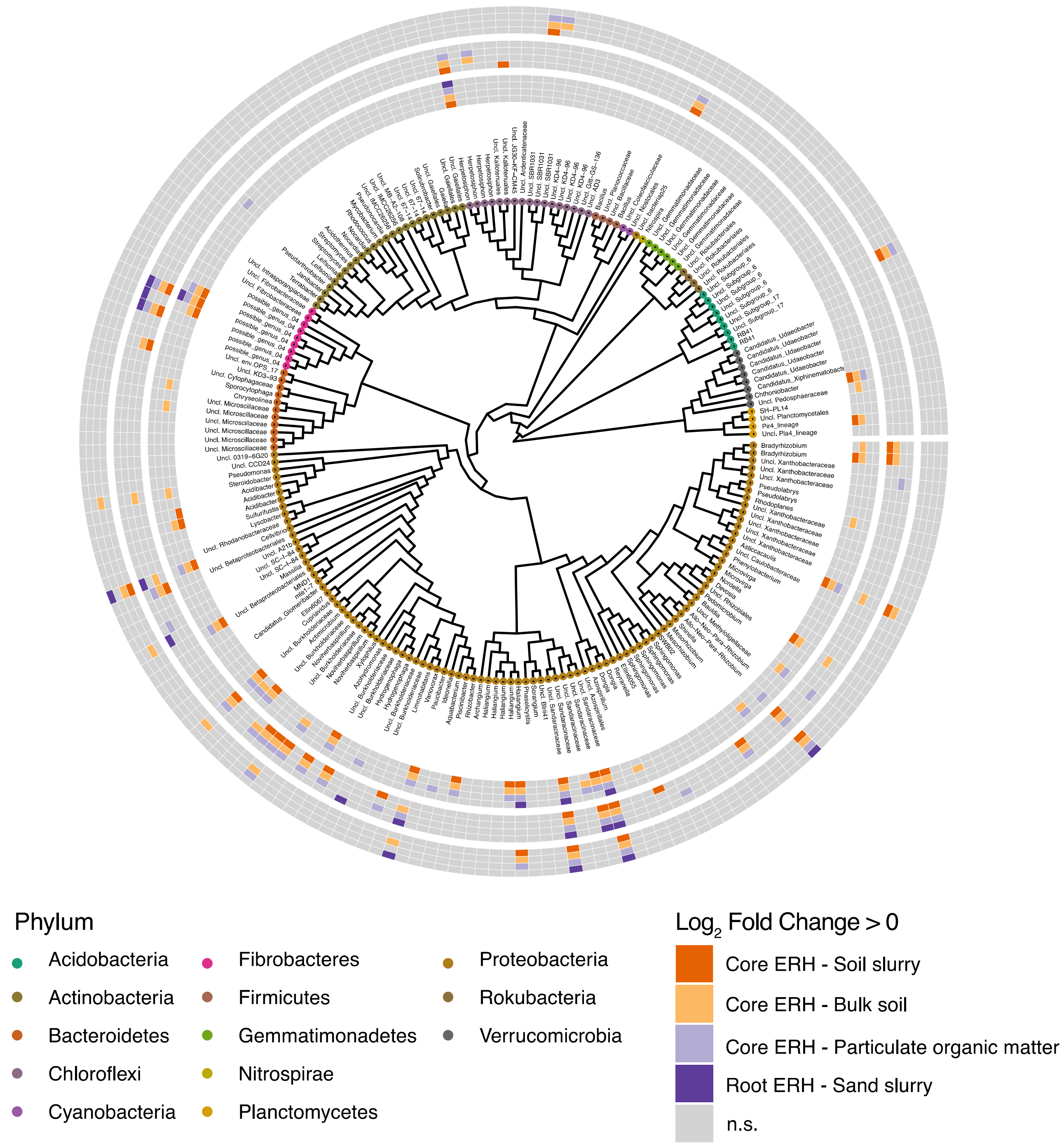

Fig. S5: Phylogenetic tree of 200 most abundant ASVs in core ERH and soil slurry samples. Heatmap highlights differentially abundant taxa enriched in ERH samples compared to multiple controls. Tip points are colored by phylum. Heatmap rings from inside: Contrasts in samples from Dryden, NY, Florence, SC and Pendleton, SC.

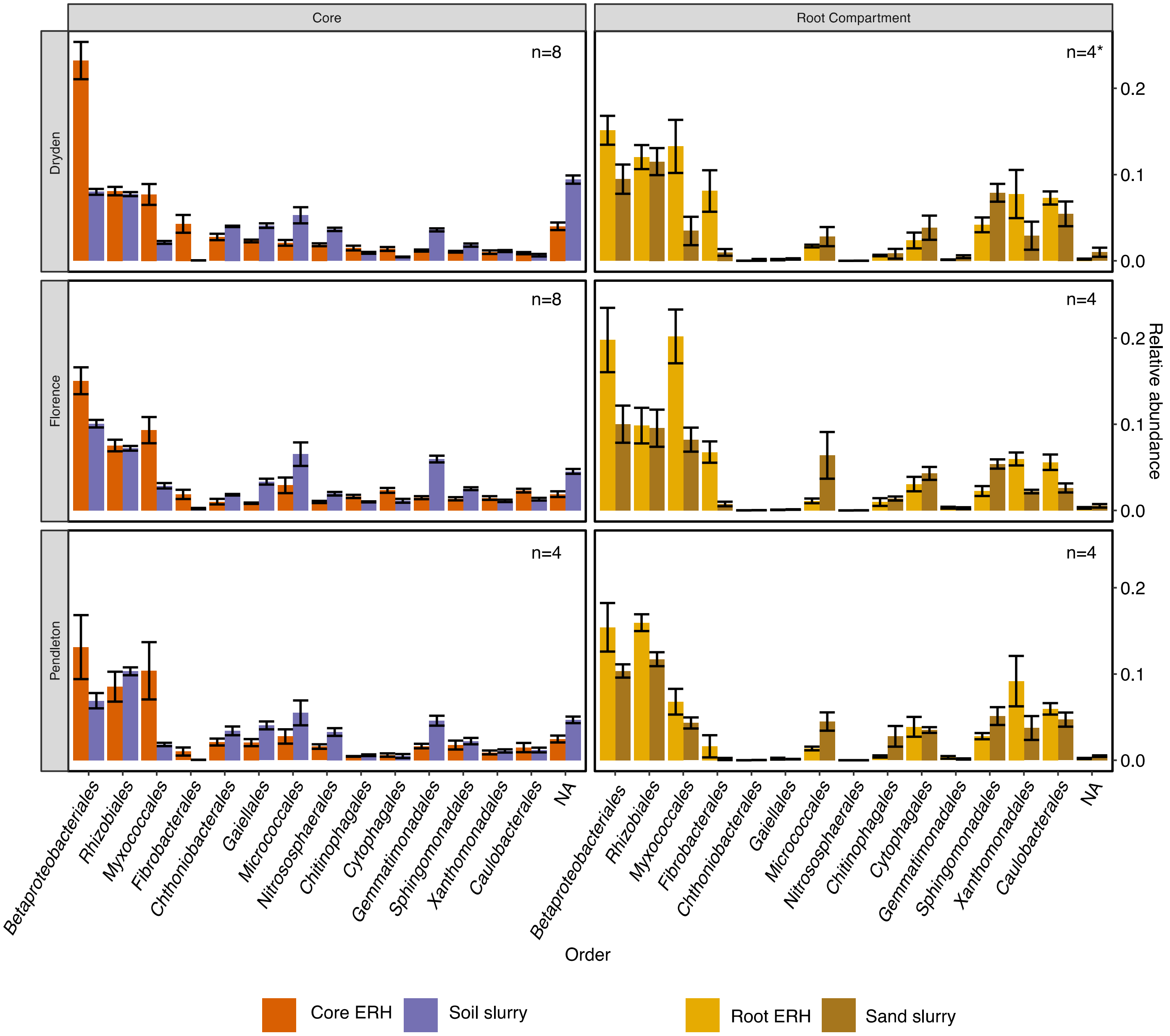

Figure S6: Community composition of ERH samples from the core (live soil-sand mixture) and root compartment (sand-gravel mixture) in Experiment 2. Relative abundance of 15 most abundant bacterial orders in ERH (orange and light brown bars) and core samples (purple and dark brown bars) are arranged by descending abundance in Dryden core ERH samples. Bars represent mean (± s.e). *Dryden root ERH samples (n = 3).

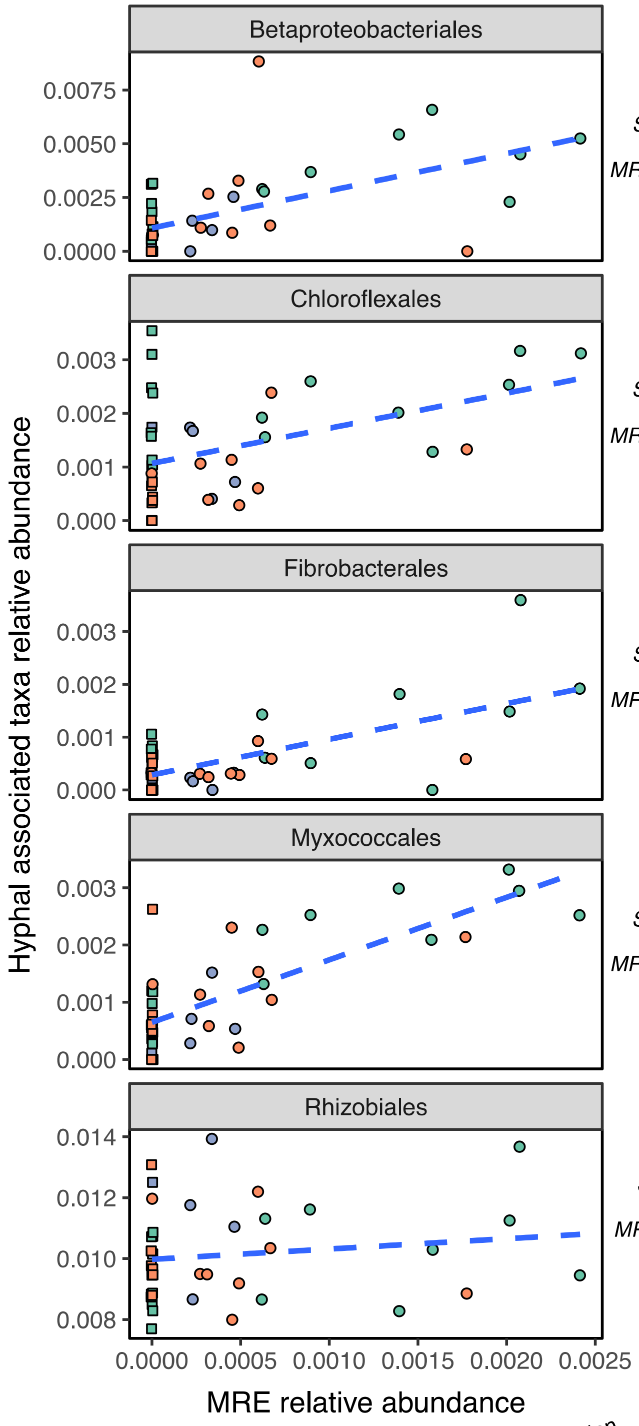

	Df	Sum Sq	Mean Sq	F value	P
Soil	2	3.4e−05	1.7e−05	6.8	0.0031
SampleType	1	4.1e−05	4.1e−05	17	0.00024
MRE rel abund	1	2e−06	2e−06	0.81	0.38
Residuals	35	8.6e−05	2.5e−06		

	Df	Sum Sq	Mean Sq	F value	P
Soil	2	1.9e−05	9.7e−06	22	7.9e−07
Sample type	1	1.4e−06	1.4e−06	3.1	0.088
MRE rel abund	1	5.4e−07	5.4e−07	1.2	0.28
Residuals	35	1.6e−05	4.5e−07		

	Df	Sum Sq	Mean Sq	F value	P
Soil	2	5.5e−06	2.8e−06	12	0.00011
Sample type	1	1.6e−06	1.6e−06	7.1	0.011
MRE rel abund	1	3e−06	3e−06	13	0.001
Residuals	35	8.1e−06	2.3e−07		

	Df	Sum Sq	Mean Sq	F value	P
Soil	2	6.9e−06	3.4e−06	9.8	0.00043
Sample type	1	1.2e−05	1.2e−05	33	1.8e−06
MRE rel abund	1	4.9e−06	4.9e−06	14	0.00072
Residuals	35	1.2e−05	3.5e−07		

	Df	Sum Sq	Mean Sq	F value	P
Soil	2	7e−06	3.5e−06	1.5	0.24
Sample type	1	4.8e−06	4.8e−06	2	0.16
MRE rel abund	1	9.4e−07	9.4e−07	0.4	0.53
Residuals	35	8.3e−05	2.4e−06		

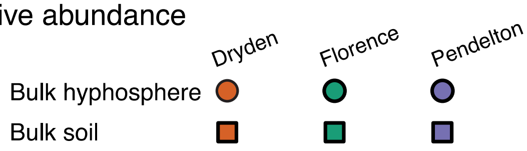

Figure S7: Correlation between hyphal colonization of soil by *G. versiforme* and summed relative abundance of hyphal associated ASVs. Relative abundance of mollicute related endobacteria (MRE) used as an indicator of hyphal colonization. Analysis of variance testing effect of soil, sample type and MRE abundance on relative abundance of each group displayed to right of panel.

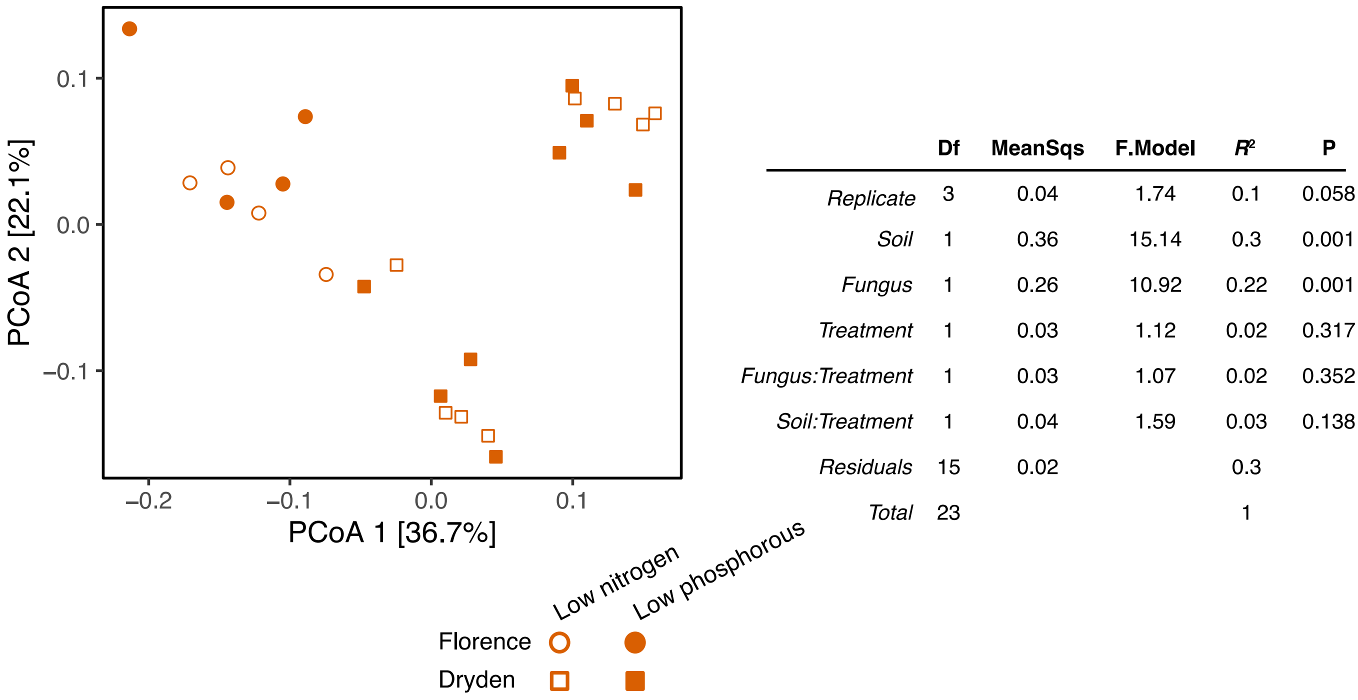

	Df	MeanSqs	F.Model	R^2	P
Replicate	3	0.04	1.74	0.1	0.058
Soil	1	0.36	15.14	0.3	0.001
Fungus	1	0.26	10.92	0.22	0.001
Treatment	1	0.03	1.12	0.02	0.317
Fungus:Treatment	1	0.03	1.07	0.02	0.352
Soil:Treatment	1	0.04	1.59	0.03	0.138
Residuals	15	0.02		0.3	
Total	23			1	

Fig. S8: Principal coordinate analysis of weighted-Unifrac distances displays variation in hyphal associated bacterial communities among soil types, without apparent variation attributed to low phosphorous (closed circles) or low nitrogen (open circles) fertility treatments. Permutational multivariate analysis of variance partitioning beta-diversity among soil, fungal species, nutrient treatment and interactions displayed to right of panel.

Supplementary Text:

16S rRNA gene amplicon library preparation and processing

For Experiment 1, barcoded libraries were prepared using the forward (515F) and reverse (806R) primers from Caporaso et al. [2] at the University of Minnesota Genomics Center (Minneapolis, MN) with v3 chemistry in the 2 x 250bp mode. Libraries from Experiments 2 and 3 were prepared following Kozich et al. [3] and sequenced on the Illumina MiSeq system at the Cornell Biotechnology Resource Center Genomics Facility (Ithaca, NY) with v2 chemistry in 2 x 250 bp mode. Briefly, triplicate reactions were prepared with ≤ 5ng DNA template, 12.5 µl of 2x Q5 High Fidelity Hot Start PCR Mastermix, 1µM combined forward and reverse primer, 0.5 µg bovine serum albumin and 0.625 µl of 4x PicoGreen reagent to monitor DNA template production in 25 µl volume with the following PCR conditions: 95°C for 2 min; 30 cycles of 95 ^{o}C for 20 s, 55 °C for 15 s and 72 °C for 10 s; final extension 72 °C for 5 min. Each library included DNA extraction blanks and PCR blanks as negative controls and mock communities of known composition. Reactions were pooled and normalized with the SequalPrep Normalization Plate Kit (Life Technologies, Carlsbad, CA), pooled and gel purified using the E.Z.N.A Gell Extraction Kit (Omega Bio-Tek, Norcross, GA).

Demultiplexed files from each library were processed separately with the 'DADA2' pipeline in R [4] to trim sequences, perform quality filtering to remove sequences with ambiguous base calls and sequences with max expected errors greater than 2, infer amplicon sequence variants (ASVs) in each sample, remove chimeras and construct a sequence count table. Taxonomy was assigned using the Silva reference database (v.132) [5]. The dataset was filtered in 'phyloseq' [6] to remove ASVs without three reads in at least three samples. Retained sequences were aligned using ssu_align [7] and bacterial phylogenetic tree was created and

rooted to *Sulfolobus* (acc. X90478) using FastTree [8] with default settings. The count table, phylogenetic tree, taxonomy table and sample metadata were combined in a ‘phyloseq’ object for further analysis. Hyphal samples were comparatively low biomass (core ERH samples yielded approximately 15.5 ±8.3 ng DNA per sample) and therefore could be susceptible to contaminating sequences during extraction and amplification. Likely contaminants were identified as those sequences that appeared in more than one extraction or PCR blank and were not abundant in high biomass samples, did not display a biological signal or displayed distinct batch effects based on extraction or amplification processing. Identified contaminants were removed from further analysis. Sequences removed included clear contaminants in one plate of extractions from Experiment 3, the most abundant of which were annotated as *Phyllobacterium* and *Sphingomonas* (*Alphaproteobacteria*). The source of contamination was not identified. Three MiSeq runs resulted in a total of 30 355 870 reads. Following quality filtering and processing this resulted in an average of 24 839 (±8 782), 40 909 (±16 882) and 46 415 (±15 236) reads in Experiment 1, 2 and 3, respectively (Supplementary Data).

Fluorescent *in situ* hybridization

Harvested hyphae were fixed overnight in 1% formalin in PBS solution, rinsed and stored in 50% EtOH in PBS solution. Fixed hyphae were mounted on gelatin coated deepwell slides. The samples were hybridized with Eubacterial probe EUB338 [9] attached to Alexa Fluor 594 in 30% formamide solution as in Manz *et al.* [10]. In addition, the *Yersinia pestis* specific probe YersPest1523 [11] was used as a negative control to confirm the absence of non-specific binding. Samples were observed under a Leica TCS-SP5 confocal microscope (Leica Microsystems Exton, PA USA).

Evaluation of sand-only controls

In Experiment 1, mesocosms were established with cores filled only with autoclaved play sand to assess the composition of a community established in the absence of a live soil inoculum. As expected, observed richness in sand controls was drastically lower compared to the live soil mesocosms (Fig. S2). While the substrate was initially sterilized, mesocosms were not established or maintained under aseptic conditions, therefore some microbial diversity was expected and may result from imperfect sterilization of the substrate, imperfect sterilization of inoculated spores, reverse osmosis H_2O used for watering, or colonization from live soil mesocosms maintained in the same growth chamber. However, results indicate that the community established on hyphae in the sand mesocosms was distinct from the hyphal communities observed in the live soil cores (Fig. S3A). Moreover, taxa abundant on the hyphae in live soil were only sporadically observed on hyphae from the sand only mesocosms (Fig. S3B). This result suggests colonization from live soil mesocosms as the most likely explanation as carryover from inoculum, substrate or watering would be expected to lead to more consistent colonization of the sand mesocosms.

1. Griffiths RI, Whiteley AS, O'Donnell AG, Bailey MJ. Rapid method for coextraction of DNA and RNA from natural environments for analysis of ribosomal DNA- and rRNA-based microbial community composition. *Appl Environ Microbiol* 2000; **66**: 5488–5491.
2. Caporaso JG, Lauber CL, Walters WA, Berg-Lyons D, Lozupone CA, Turnbaugh PJ, et al. Global patterns of 16S rRNA diversity at a depth of millions of sequences per sample. *PNAS USA* 2011; **108**: 4516–4522.

3. Kozich JJ, Westcott SL, Baxter NT, Highlander SK, Schloss PD. Development of a dual-index sequencing strategy and curation pipeline for analyzing amplicon sequence data on the MiSeq Illumina sequencing platform. *Applied and Environmental Microbiology* 2013; **79**: 5112–5120.

4. Callahan BJ, McMurdie PJ, Rosen MJ, Han AW, Johnson AJA, Holmes SP. DADA2: High-resolution sample inference from Illumina amplicon data. *Nature Methods* 2016; **13**: 581–583.

5. Quast C, Pruesse E, Yilmaz P, Gerken J, Schweer T, Yarza P, et al. The SILVA ribosomal RNA gene database project: improved data processing and web-based tools. *Nucl Acids Res* 2013; **41**: D590–D596.

6. McMurdie PJ, Holmes S. phyloseq: An R package for reproducible interactive analysis and graphics of microbiome census data. *PLoS ONE* 2013; **8**: e61217.

7. Nawrocki EP. Structural RNA homology search and alignment using covariance models. [dissertation]. [St. Louis (MO)]: Washington University School of Medicine. 2009. Ph.D, Washington University School of Medicine.

8. Price MN, Dehal PS, Arkin AP. FastTree: Computing large minimum evolution trees with profiles instead of a distance matrix. *Mol Biol Evol* 2009; **26**: 1641–1650.

9. Amann RI, Devereux R, Stahl' DA. Combination of 16S rRNA-targeted oligonucleotide probes with flow cytometry for analyzing mixed microbial populations. *Applied and Environmental Microbiology* 1990; **56**: 7.

10. Manz W, Amann R, Ludwig W, Wagner M, Schleifer K-H. Phylogenetic oligodeoxynucleotide probes for the major subclasses of Proteobacteria: Problems and solutions. *Systematic and Applied Microbiology* 1992; **15**: 593–600.

11. Rohde A, Hammerl JA, Appel B, Dieckmann R, Al Dahouk S. Differential detection of pathogenic *Yersinia* spp. by fluorescence in situ hybridization. *Food Microbiology* 2017; **62**: 39–45.
